# Analysis of the Incidence of Ocular Extraintestinal Manifestations in Inflammatory Bowel Disease Patients: A Systematic Review

**DOI:** 10.3390/diagnostics14242815

**Published:** 2024-12-14

**Authors:** Bruno Songel-Sanchis, Jesús Cosín-Roger

**Affiliations:** Departamento de Farmacología and CIBERehd, Facultad de Medicina, Universidad de Valencia, Av. Blasco Ibáñez, 15, 46010 Valencia, Spain; bruson@alumni.uv.es

**Keywords:** inflammatory bowel disease, ocular extraintestinal manifestations, incidence, uveitis, episcleritis, scleritis

## Abstract

**Background**: Inflammatory bowel disease (IBD), which includes Crohn’s disease and ulcerative colitis, primarily affects the gastrointestinal tract. Additionally, extraintestinal manifestations may occur in the liver, musculoskeletal system and eyes. Its etiology remains unknown, and further research is required in order to develop pharmacological drugs which achieve complete remission of the pathology. **Objective**: The aim of this study was to analyze the incidence of ocular extraintestinal manifestations in IBD patients. **Methods**: A total of six searches were carried out on the medical publication server “*PubMed*” in June and July 2024, using different keywords; a total of 323 results were obtained, of which 34 were finally selected for study. **Results**: Ocular extraintestinal manifestations in IBD patients are more common in the anterior pole of the eye, with uveitis, scleritis and episcleritis being the most usual ones. In the case of the posterior pole, the most common manifestations are posterior uveitis and optic neuritis. **Conclusions**: The incidence of ocular complications whose origin is inflammatory, such as uveitis, scleritis, episcleritis and neuritis, is higher than that of complications of non-inflammatory origin.

## 1. Introduction

Inflammatory bowel disease (IBD) is a complex multifactorial condition that includes two pathologies: Crohn’s disease (CD) and ulcerative colitis (UC). Both share clinical and pathophysiological characteristics but present differences in the location and extent of the inflamed area [1,2]. Crohn’s disease primary affects a defined area, most commonly in the lower intestine and colon. These signs can appear in any segment of the gastrointestinal tract, from the mouth to the anus. On the other hand, in ulcerative colitis patients, the inflammation is limited to the colon, showing a more extensive distribution and the possibility of developing ulcers in the mucosa with a more diffuse pattern and less delimited area than in Crohn’s disease, accompanied by inflammatory signs [3,4,5]. Several factors participate in their development, including environmental and genetic factors and the patient’s own intestinal microbiome, which interacts in a complex way, triggering chronic inflammatory responses in the intestine [1,6]. The characteristic symptoms of the disease are, among others, abdominal pain, chronic diarrhea, rectal bleeding, weight loss, fatigue, fever and lack of appetite [7].

The pathophysiology of IBD focuses on the intestinal epithelium, a monolayer of cells that lines the interior of the intestine and is in contact with the luminal contents. Under normal conditions, this layer of epithelial cells, which forms a physical barrier, and the cells of the immune system maintain a balance that allows for intestinal homeostasis. However, in IBD patients, several alterations impair the integrity of the epithelial barrier, allowing for the penetration of bacteria from the saprophytic flora into the underlying tissues. This bacterial invasion triggers a deregulated and excessive immune response, in which T cells, macrophages, neutrophils, immunoglobulins G and dendritic cells participate, thus contributing to the perpetuation of chronic inflammation [3].

Over the last 50 years, there has been a significant increase in the number of IBD patients. All the studies published to date have made it possible to identify molecular mechanisms involved in the pathology and have contributed to the development of new pharmacological therapies. However, despite these advances, IBD is still considered an unknown disease in terms of medical knowledge. The exponential increase in patients affected by IBD constitutes a challenge for health systems worldwide. In fact, it is estimated that in recent decades, CD patients have increased by 75% and 60% in the case of UC patients [8]. This upward trend has generated great concern, as it has become a public health problem. The need for adequate resources and strategies for the diagnosis, management and follow-up of patients with IBD has become increasingly pressing [2,3]. It is important to highlight that the epidemiological data of IBD are not well defined nor categorized, due to its recent increase and lack of research. However, it is being concluded that the development of technology, together with the appearance of new lifestyles that involve stress and unbalanced diet characterized by high-fat, high-sugar, and low-fiber content, causes the environmental factor to develop, favoring the appearance of this disorder, since it has been observed that its prevalence is much higher in developed countries and in young adults, who are increasingly subject to lifestyles that include these habits that negatively affect this disease [9,10].

During the disease, and even before its diagnosis, it is common to observe the appearance of extraintestinal manifestations (EIMs) derived from IBD. In fact, there are several authors who refer to IBD as a systemic disease that mainly affects the gastrointestinal tract. These complications have a significant impact on the skin, liver, musculoskeletal system and eye [11]. It is estimated that their origin is related to the release of cytokines, which promote inflammation and, in some cases, can spread to these tissues [12]. In this scenario, it is important to highlight that despite not having very high incidence, some patients suffering from IBD develop ocular extraintestinal manifestations that compromise their already low quality of life. Dry eye, scleritis, episcleritis and uveitis are the most common ocular complications in patients with IBD, where all are of inflammatory origin [11]. Each of these conditions can cause discomfort, pain, and visual impairment. Dry eye is a chronic eye condition characterized by decreased tear production or quality, resulting in insufficient lubrication and causing symptoms such as itching, redness and foreign body sensation. This condition can be caused by various factors such as aging, exposure to dry or air-conditioned environments, prologued use of digital screens, certain medications and tear gland problems. Treatment for this complication is administered by using artificial tears, lubricating ointments, anti-inflammatory medications or topical immunomodulators [13]. Scleritis is an inflammatory eye disease characterized by inflammation of the sclera, which is the tough white layer that covers the eye. This condition can be very painful and affect vision, if not treated properly. Patients with IBD and scleritis often experience severe eye pain, redness, sensitivity to light and decreased vision. Treatment of this manifestation generally includes the use of anti-inflammatory drugs such as topical and systemic corticosteroids, taking into account the risk of developing glaucoma or cataracts. In more severe cases, immunosuppressive or biological medications may be required. In addition to medications, complementary measures may be recommended, such as eye protection against intense sun exposure [14]. Episcleritis is an inflammatory eye disease that affects the layer between the sclera and the conjunctiva, which is a thin, vascularized structure. Although it is not a serious condition, it can cause severe discomfort. Episcleritis associated with IBD presents as eye redness, irritation, high sensitivity to light and foreign body sensation. Treatment of the complication is based on administering anti-inflammatory eye drops, such as topical steroids, with the aim of relieving ocular inflammation. In addition to pharmacological treatment, there are other measures that can help in the management of episcleritis, such as applying cold patches to the affected eye, which can provide relief and reduce localized inflammation. It is also recommended to avoid the use of contact lenses during the inflammatory episode, as they may increase irritation and prolong recovery [15]. Uveitis is a serious inflammation of the uveal tract, which includes the iris, ciliary body and choroid [16].

Changes in corneal and retinal thickness have been also described [17,18]. In fact, it has been described that some patients can have a thinner cornea and thicker peripheral retina. This can be a risk factor for many pathologies or surgical procedures, as well as a biomarker for the diagnosis of IBD.

Given the presence of extraintestinal manifestations in IBD patients and the scarce literature analyzing the incidence of, specifically, ocular manifestations, the main objective of this systematic review is to evaluate the currently available data on the incidence of those extraintestinal manifestations that affect specifically the eye.

## 2. Methods

This systematic review was conducted according to the Preferred Reporting Items for Systematic Reviews and Meta-Analyses (PRISMA) guidelines [19]. When we wrote the systematic review, we used the PRISMA 2020 checklist in order to ensure compliance with the PRISMA 2020 statement. This checklist has also been submitted as Supplementary Information.

A total of 6 comprehensive literature searches were conducted across the database PubMed during the months of June and July 2024. These searches were made by using the following keywords: “Ocular complications and IBD”, “Ocular manifestations and IBD”, “Ocular complications and IBD incidence”, “Ocular manifestations and IBD incidence”, “Ocular complications and IBD treatment” and “Ocular manifestations and IBD treatment”.

The inclusion criteria for this systematic review encompassed original research articles reporting ocular manifestations in IBD patients between January 2010 and June 2024. The exclusion criteria were editorials, bibliographic reviews, animal studies without human data, conference abstracts, non-English articles, those which did not describe the incidence nor clinical information about the patients included in the study and articles lacking a direct connection with the main objective of this systematic review.

**Figure 1:**
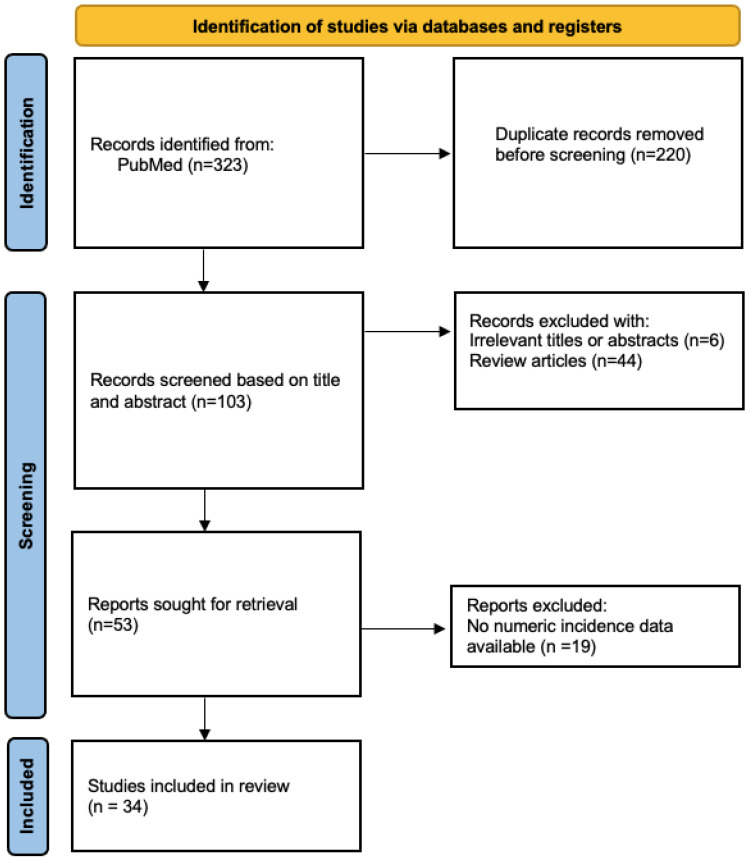
The PRISMA flow diagram for systematic reviews showing the process used to identify the papers included in the systematic review.

After these searches, a total of 323 articles were obtained, of which 220 were removed before screening due to duplicate records and 69 were discarded due to the following reasons: a total of 6 records were not related to the topic to be discussed according to the title and abstract, 19 did not provide useful numeric incidence data for the research study, and 44 were bibliographic reviews. Therefore, 34 articles were finally selected for analysis (Figure 1).

The quality of the studies included in this systematic review was scored by two independent researchers (B.S.-S. and J.C.-R.) by using the Newcastle Ottawa Scale (NOS) adjusted for case–control studies. This scale has a score ranging from 0 to 9 points based on three domains: selection, comparability and outcome. The studies were rated from 0 to 9 and subdivided according to the score into poor quality (0–2 points), fair quality (3–5 points) and good/high quality (6–9 points) [20]. All the studies included in this systematic review received 6 or more points, indicative of their good and high quality.

## 3. Results and Discussion

The incidence of ocular extraintestinal manifestations in IBD described in the literature covers a wide range of cases and presents significant variability. Although these ocular complications are rare in percentage terms, their importance cannot be underestimated, and it is essential to describe them in detail in this section. To facilitate its understanding, the results that show ocular involvement without specifying the type are shown first in Table 1. Secondly, the results have been grouped according to the affected ocular region: posterior pole (Table 2) and anterior pole (Table 3).

As shown in Table 1, a study comparing samples of different races in several countries (Caucasians, Asians born in the United States and Asians living in United States) reported different percentages of the incidence of O-EIMs. Specifically, approximately half of the Asian population included in the study presented ocular complications, whereas a lower percentage in the Caucasian population was reported. At this point, it is worth mentioning the significant difference in the sample size, which might explain these differences. Therefore, additional studies are needed in order to further analyze the incidence of these O-EIMs according to the race of IBD patients.

The rest of the studies included in this systematic review reported that 3.6%, 20%, 0.5%, 3.2%, 2%, 11.6%, 7.1%, 4%, 3% and 9.8% of all patients analyzed developed ocular extraintestinal manifestations. The second highest incidence of these manifestations was shown in the USA, which probably may be related to the lifestyle in this country, more becoming to the development of the disease. In line with these results, several studies have reported an alarming increase in the incidence of IBD in developed countries [21,22]. Nevertheless, the sample size of this study was not the biggest population included in one study, which highlights the need to confirm whether this higher incidence would be reproduced in a bigger cohort of patients living in the USA.

**Table 1:** Incidence of unspecified ocular extraintestinal manifestations of IBD.

Reference	Country	N (IBD)	N (UC)	N (CD)	O-EIMs	Affected by IBD (%)	Affected by UC (%)	Affected by CD (%)
[23]	USA				UN			
	White	5223	UN	UN		37 (0.7)	UN	UN
	Asians born in the USA	35	UN	UN		14 (40)	UN	UN
	Asians living in the USA	81	UN	UN		41 (51)	UN	UN
[24]	USA	3452	UN	UN	UN	124 (3.6)	UN	UN
[25]	Egypt	200	UN	UN	UN	40 (20)	UN	UN
[26]	Spain	31,077	UN	UN	UN	150 (0.5)	UN	UN
[27]	Italy	811	595	216	UN	26 (3.2)	10 (4.6)	16 (7.4)
[28]	Israel	10,260	UN	UN	UN	199 (1.9)	UN	UN
[29]	USA	757	269	488	UN	88 (11.6)	17 (6.3)	71 (15)
[30]	USA	4108	UN	UN	UN	292 (7.1)	UN	UN
[31]	USA	12,083	UN	UN	UN	483 (4)	UN	UN
[32]	Italy—Holland	202	UN	UN	UN	6 (3)	UN	UN
[33]	Denmark	32,446	22,144	10,302	UN	3180 (9.8)	1363 (6.2)	1817 (18)

N: number of patients included in the study. O-EIMs: ocular extraintestinal manifestations. IBD: inflammatory bowel disease. UC: ulcerative colitis. CD: Crohn’s disease. UN: unspecified.

Following the above-described studies showing the incidence of ocular manifestations without stating the specific O-EIM developed, the studies describing ocular manifestations which affect specifically the posterior pole are summarized in Table 2.

The O-EIMs found in these studies were mainly central serous chorioretinopathy, optic neuritis, serous retinal detachment, ischemic optical neuropathy, posterior uveitis and hypertonic fundus. The incidence rates in percentage were 1.2% for central serous chorioretinopathy, 0.2% for optic neuritis, 1.6% for serous retinal detachment, 0.2% for ischemic optical neuropathy, 0.4% for posterior uveitis and 13.1% for hypertonic fundus. It is important to highlight that among the different ocular complications affecting the posterior pole, hypertonic fundus is the most frequent compared with the rest of the complications, with a big difference. This sign is related to hypertensive retinopathy, a common pathology associated with arterial hypertension, also associated with high levels of stress. These results point to the fact that specifically, hypertonic fundus is the most frequent complication affecting the posterior pole in IBD patients.

Also, a case report described orbital myositis in one IBD patient. Orbital myositis is a general term for inflamed extraocular muscles whose causes, among others, are autoimmune diseases such as IBD [34]. A remarkable aspect of this case report is the fact that this complication was directly related and taken as the initial sign before gastrointestinal manifestations. Although this O-EIM is a rare manifestation of IBD and even more rarely occurs in the initial presentation, even before the development of IBD, it is important to further analyze this complication as a possible symptom that occurs before IBD.

**Table 2:** Incidence of ocular extraintestinal manifestations in IBD in posterior pole.

Reference	Country	N (IBD)	N (UC)	N (CD)	O-EIMs	Affected by IBD (%)	Affected by UC (%)	Affected by CD (%)
[35]	France	87	UN	UN	Central serous corioretinopathy	1 (1.2)	UN	UN
[36]	Taiwan	4505	UN	UN	Optic neuritis	8 (0.2)	UN	UN
[37]	Corea	61	25	36	Optic neuritis	1 (1.6)	0	1 (2.8)
		61	25	36	Serous retinal detachment	1 (1.6)	0	1 (2.8)
[38]	Taiwan	4508	UN	UN	Ischemic optical neuropathy	7 (0.2)	UN	UN
[39]	Greece	1860	859	1001	Posterior uveitis	7 (0.4)	4 (0.5)	3 (0.3)
[40]	Germany	61	UN	UN	Hypertonic fundus	8 (13.1)	UN	UN
[41]	USA	1	UN	UN	Orbital myositis	1 (100)	UN	UN

N: number of patients participating in the study. O-EIMs: ocular extraintestinal manifestations. IBD: inflammatory bowel disease. UC: ulcerative colitis. CD: Crohn’s disease. UN: unspecified.

After describing the complications which affect the posterior pole, in Table 3, the studies reporting complications affecting the anterior pole are included. Cataracts represented 13.5%, 62.3%, 2.1% and 10.4% of all the cases included in the studies. In spite of the fact that the values are quite variable, most of the studies stated that this complication might be more related to the corticosteroid treatment rather than to the age of IBD patients, since the incidence of this complication was not different among the different populations included in all the studies (specifically, the mean ages were 48.05, 43.3, 11.25 and 31 years, respectively). Moreover, given the well-known side effects of corticoids, among which are cataracts [42], it is reasonable to assume that most of the cataracts developed in IBD patients might be due to long-term treatment with these pharmacological drugs.

Uveitis represented 4.2%, 0.9%, 2.3%, 1.2%, 1.1%, 23.6%, 1.7%, 1.2%, 3.2%, 2.9%, 3.1%, 0.1% and 15% of the cases studied. The results show lower incidence of this O-EIM, except for the 23.6% study, in which it was concluded that the classification of uveitis was not clear and specified. Therefore, the results point to the fact that this complication is not very common in IBD patients.

Regarding the rest of the O-EIMs affecting the anterior pole, dry eye represented the highest rates of incidence, specifically 12.5%, 6.3%, 68.9%, 42.1% and 17.2% of the cases studied. This ocular disorder is characterized by enhanced osmolarity of the tear film and the activation of the inflammatory pathways on the ocular surface. Patients with this complication suffer from several symptoms, such as itching, blurred vision and burning, which diminishes their quality of life [43]. Although the association between IBD and the ocular manifestation dry eye is still unclear, the results suggest that the incidence of this disorder is significantly higher in non-IBD patients compared with IBD patients. On the other hand, it is important to note that the rest of the complications exhibited lower incidence. In fact, iridocyclitis represented 1.8% and 2.2% of the cases studied, whereas scleritis was represent in 1%, 2.7%, 0.6%, 0.2% and 2.3% of the patients analyzed. In the case of episcleritis, it affected 0.6%, 1%, 1.6%, 15%, 0.9%, 1% and 5.8% of the cases studied, while conjunctivitis was present in 1.5% and 4.6% of the total of subjects analyzed. At this point, it is worth mentioning that in line with other ocular disorders caused by the activation of the inflammatory pathways, these previous complications affecting the anterior pole whose origin is also inflammatory have been found increased in IBD patients. Finally, corneal surface injures affected 1.9% of the cases studied, and peripheric ulcerative keratitis affected 0.2% and 1,2% of the IBD patients included in the studies. In addition, another case reporting ptosis, which was also subsequent of the diagnosis of IBD, is also included in this table.

**Table 3:** Incidence of ocular extraintestinal manifestations in IBD in anterior pole.

Reference	Country	N (IBD)	N (UC)	N (CD)	O-EIMs	Affected by IBD (%)	Affected by UC (%)	Affected by CD (%)
[44]	Italy	96	UN	UN	Uveitis	4 (4.2)	UN	UN
					Dry eye	12 (12.5)	UN	UN
					Cataracts	13 (13.5)	UN	UN
[45]	Hungary	508	205	303	Iridocyclitis	9 (1.8)	5 (2.4)	4 (1.3)
					Scleritis	5 (1)	3 (1.5)	2 (0.7)
[33]	Denmark	32,446	22,144	10,302	Iridocyclitis	714 (2.2)	292 (1.3)	422 (4.1)
[46]	Corea	531	UN	UN	Uveitis	5 (1)	UN	UN
					Episcleritis	3 (0.6)	UN	UN
[47]	Turkey	44	UN	UN	Uveitis	1 (2.3)	UN	UN
[13]	Taiwan	54,293	UN	UN	Dry eye	3421 (6.3)	UN	UN
					CSI	1003 (1.9)	UN	UN
[48]	India	1449	1146	303	Episcleritis	14 (1)	10 (0.9)	4 (1.3)
					Uveitis	18 (1.2)	16 (1.4)	2 (0.7)
[49]	Italy	92	46	46	Uveitis	1 (1.1)	0	1 (2.2)
[37]	Corea	61	24	36	Iritis	3 (4.9)	1 (4.2)	2 (5.6)
					Episcleritis	1 (1.6)	0	1 (2.8)
[50]	United Kingdom	93,796	UN	UN	Uveitis	22,098 (24)	UN	UN
					Episcleritis	13,955 (15)	UN	UN
					Scleritis	2482 (2.7)	UN	UN
[39]	Greece	1860	805	1001	Episcleritis	16 (0.9)	0	16 (1.6)
					Uveitis	31 (1.7)	6 (0.8)	25 (2.5)
[40]	Germany	61	UN	UN	Dry eye	42 (69)	UN	UN
					Cataracts	38 (62)	UN	UN
					Blepharitis	26 (43)	UN	UN
[51]	Hungary	873	619	254	Uveitis	10 (1.2)	6 (1)	4 (1.6)
					Conjunctivitis	13 (1.5)	9 (1.5)	4 (1.6)
					Scleritis	5 (0.6)	4 (0.7)	1 (0.4)
[52]	USA	1320	UN	UN	Uveitis	42 (3.2)	UN	UN
					Scleritis	2 (0.2)	UN	UN
					PUK	2 (0.2)	UN	UN
[53]	Sweeden	483	330	153	Uveitis	14 (2.9)	13 (3.9)	1 (0.7)
[54]	Brazil	88	UN	UN	Dry eye	37 (42)	UN	UN
[55]	Iran	96	UN	UN	Cataracts	2 (2.1)	UN	UN
					Uveitis	3 (3.1)	UN	UN
					Episcleritis	1 (1)	UN	UN
[56]	Italy	3952	2109	1843	Uveitis	5 (0.1)	1 (0.1)	4 (0.2)
[57]	USA	1211	425	786	Uveitis, iritis	37 (3.1)	7 (1.7)	30 (3.8)
[35]	France	87	UN	UN	Dry eye	15 (17)	UN	UN
					Uveitis	13 (15)	UN	UN
					Episcleritis	5 (5.8)	UN	UN
					Scleritis	2 (2.3)	UN	UN
					PUK	1 (1.2)	UN	UN
					Blepharitis	9 (10)	UN	UN
					Conjunctivitis	4 (4.6)	UN	UN
					Cataracts	9 (10)	UN	UN
[58]	Spain	1	UN	UN	Ptosis	1 (100)	UN	UN

N: number of patients participating in the study. O-EIMs: ocular extraintestinal manifestations. IBD: inflammatory bowel disease. UC: ulcerative colitis. CD: Crohn’s disease. CSI: corneal surface injury. PUK: peripheric ulcerative keratitis. UN: unspecified.

Taking into account all the results obtained, the ocular extraintestinal manifestations of IBD patients are more common in the anterior pole of the eye, with dry eye, uveitis, scleritis and episcleritis being the most usual complications. On the other hand, in the case of the posterior pole, the most common manifestations are posterior uveitis and optic neuritis. It is important to highlight that the most common extraintestinal ocular manifestations of IBD are logically those of inflammatory origin, such as uveitis, scleritis, episcleritis or neuritis. Moreover, cases of cataracts are also observed and may be related to the use of corticosteroids applied to treat the symptoms of IBD. Cases of ulcerative peripheral keratitis, caused by the increase in cytokines at a systemic level and their cytotoxic damage to the tissues, have also been described.

Another important aspect which needs to be highlighted is that the incidence of eye disorders derived from IBD is higher in developed countries, possibly due to two factors: (a) the lack of studies in developing countries and (b) the lifestyle and conditions in developed countries, which favor the development and appearance of the disease in these countries. Furthermore, although there are no differences in the incidence of ocular manifestations between Crohn’s disease and ulcerative colitis patients in the studies included in this systematic review, it is important to state that the results are conditioned mainly by the sample size. In addition, in many studies, there are no indications about the type of IBD, which makes it more difficult to analyze the incidence separately depending on the pathology. (Also, in those where the type of IBD was mentioned, there was not a clear tendency line which could determine results for the incidence of UC and CD separately.)

This study presents some limitations derived not only from the methodology previously described but also from the studies reported to date. It is important to note the potential biases might compromise the credibility and validity of this systematic review. Probably due to the low incidence of O-EIMs in IBD patients, scarce research has been published focused on these specific ocular extraintestinal manifestations, whereas most of the studies have reported extraintestinal manifestations affecting different parts of the gastrointestinal tract, such as the liver or the musculoskeletal system. In fact, we detected an imbalance between the number of studies analyzing ocular extraintestinal manifestations and those on other affected organs. Additional studies are definitively needed in order to study the incidence of these specific ocular complications in IBD patients more in depth. In addition, the lack of studies including a big cohort of patients increases the variability of the values of the percentage of incidence obtained from several studies with smaller cohorts of patients.

Another limitation we have found in this study is the lack of some clinical information we consider extremely important for this study. For instance, several studies did not differentiate between UC and CD, which made it impossible for us to compare these subtypes of IBD. We strongly believe that it would be very interesting to analyze the incidence of O-EIMs specifically in UC and CD separately, based on the considerable differences between the two diseases. In addition, we also detected differences in the number of published studies at the geographical level. In fact, most of the studies have been performed in more developed countries in Europe, North America and Asia, whereas there are a scarce number of studies analyzing IBD patients in developing countries from South America or Africa. These notable differences in the reported studies prevent us from knowing the incidence of O-EIMs worldwide.

Nevertheless, in spite of these limitations, due to the full transparency of the methodology employed and the selection of all the studies included in this systematic review, we would like to highlight that this review is absolutely objective, informative and rigorous.

## 4. Conclusions

IBD patients suffer from several extraintestinal manifestations, which can also affect the eye. Indeed, ocular extraintestinal manifestations have been reported in these patients affecting both the anterior and posterior poles. Regarding the incidence of these complications, it is important to state that the most frequent O-EIMs are those of inflammatory origin affecting the anterior pole, such as uveitis, scleritis, episcleritis and neuritis. Future studies are needed in order to better know the incidence of the rest of the ocular manifestations and to elucidate whether these incidence rates are higher in UC or CD patients, since scarce research has been published with this objective. In addition, another aspect which is important to consider is the fact that the pharmacological treatment used in these patients chronically can also trigger the development of some ocular complications. For instance, the use of corticoids might increase the incidence of cataracts in IBD patients. This aspect reflects that O-EIMs can develop not only from the pathophysiology of IBD directly but also as a consequence of a side effect derived from the pharmacological drugs administered to these patients. Therefore, despite the variability of the O-EIMs reported to date, a deeper analysis of these complications represents a milestone which needs to be addressed with further studies. A better understanding of these complications might considerably improve the quality of life of IBD patients.

## Data Availability

No new data were created or analyzed in this study.

## Supplementary Materials

The following supporting information can be downloaded at: https://www.mdpi.com/article/10.3390/diagnostics14242815/s1, Table S1: PRISMA 2020 checklist with all the items and their location in the systematic review.
